# Experiences from a language development intervention in nursing homes from the perspective of first-line managers, language advocates and care staff: a qualitative study

**DOI:** 10.1186/s12877-026-08354-1

**Published:** 2026-09-25

**Authors:** Eriksson Elisabet, Cecilia Arving

**Affiliations:** 1 Faculty of Health and Occupational Studies, University of Gävle, 80176 Gävle, Sweden; 2 Department of Public Health and Caring Sciences, Uppsala University, PO Box 564, 75122 Uppsala, Sweden

**Keywords:** Qualitative, Cross-cultural communication, Nursing homes, Language development intervention

## Abstract

**Background:**

An increase in foreign-born workers in nursing homes (NHs) has challenged first-line managers to implement language initiatives for their staff. Evidence regarding foreign-born care workers’ experiences of language support initiatives is still scarce. Thus, the aim of the study was to explore and describe the experiences of a language development intervention “Talk about Work” from the perspective of first-line managers (FLMs), language advocates and participating care staff.

**Methods:**

An explorative qualitative study using on-going evaluation methods following the intervention “Talk about Work.” The intervention consisted of eight workshops intended to strengthen language and professional competence within health and social care. Semi-structured focus-group interviews were conducted with FLMs (3), language advocates (5), and caring staff including language assistants (20) at NHs. Data were analyzed using qualitative content analysis to identify three main categories and seven subcategories.

**Results:**

The educational material was suitable for caring staff, and FLMs found it beneficial also for future education plans. They could repeat topics, introduce new ones, and continue the intervention at different paces. Addressing Swedish language skills at work was perceived as sensitive, and the initiative made it easier to speak about language competences. Challenges for FLMs included finding staff with the right competences to lead the initiative. Language advocates asked for closer collaboration with FLMs.

**Conclusion:**

The intervention “Talk about Work” was considered appropriate for and was well received by all participating groups in the study. Moreover, FLMs could use the material for continued language initiatives at NHs. To improve language and communication skills among caring staff in multi-cultural contexts, there is a need to find staff with the right competences to lead language development initiatives as well as to provide resources to implement them.

**Supplementary Information:**

The online version contains supplementary material available at https://doi.org/10.1186/s12877-026-08354-1.

## Introduction

To solve the problem of staff shortages in elderly care services, such as nursing homes (NHs), foreign-born care workers have been employed even if they have no previous experience or education in elderly care provision [3, 11, 20, 31] Sweden's municipalities and regions [SKR], [26]. For first-line managers (FLMs) in NHs, this is a challenge they must address [6, 14]; [27]. There is considerable knowledge regarding the experiences and needs of foreign-born workers within health care services [1, 2, 8, 16, 24]. However, studies exploring the experience of language development interventions for foreign-born care workers are scarce [6, 9]; [18]. Thus, the present qualitative research article contributes by filling some of the knowledge gap in this area by exploring the experiences among FLMs and their care workers of a language development intervention to overcome difficulties like communication and culture.

## Background

To meet the increased care demands among the elderly and to solve the problem of staff shortages, foreign-born care workers have been recruited and employed in high-income countries [3, 11, 20, 26, 31]. A foreign-born worker is defined as an individual who was not born as a citizen of the country they are residing and working in, e.g., naturalized citizens, lawful permanent residents and refugees (The U.S. Bureau of Labor Statistics [BLS], [32]. In Sweden, the population of foreign-born constitutes about 20% of the population (10 million; [25] and consists of a mix of nationalities (> 140), mainly originating from, e.g., Afghanistan, Africa, Asia, Latin America and the Middle East, in addition to foreign-born workers from Europe. This requires forming a heterogeneous and multilingual group of individuals with different cultures, ethnicities, religions and languages than the majority population of the host country. Further, in Sweden, about half of elderly care staff are foreign-born [26], and about 30% lack vocational training (e.g., nurses, nursing staff, health personnel, care staff, nursing aides) for working in elderly care—such as NHs, residential living homes and long-term care facilities, all referred to as NHs hereafter [8, 26]. Similarly, the proportion of foreign-born care staff working in England was approximately 32% [12] and 14% in Norway [3].

Although the contributions made by foreign-born care workers are significant [29], research has shown that this contribution may also be double-edged. In the review by Adebayo and colleagues [1], foreign-born elderly care workers reported discrimination and limited understanding of the workplace culture as barriers to retention. Also, in Eriksson and colleagues’ review (2023), the demanding work situation reported by foreign-born elderly care workers was attributed to acculturation stress caused by, e.g., poor language skills, intercultural relations and discrimination. Several studies have reported that poor native-language proficiency among foreign-born care workers contributed to communication difficulties between native NH residents and care workers [1, 7, 16].

To address communication difficulties between residents, native-born staff and foreign-born care workers, different interventions have been used. Krohne and colleagues [18] explored the experiences of a 12-month-long “Minority ABC-program.” The program focused on improving competence and interaction among elderly care workers with foreign-born and those with native-language backgrounds. Participants experienced increased inclusion at work and better competence in relation to a more holistic form of teamwork. Other interventions to address communication difficulties included foreign-born care workers using their smartphones to interpret residents' language and non-verbal communication [1]. Gerchow and colleagues [9] reported that only five of the 48 studies in their review looked at interventions intended to improve language barriers between nurses and patients. Four intervention studies included some sort of technical device for interpretation, and one evaluated an online communication skills training program for oncology nurses. The nurses also described feelings of being untrained and unprepared to manage language barriers effectively. They expressed a need for education and support from the FLM.

Thus, FLMs play an essential role in ensuring favorable integration of their multicultural and multilingual NH staff, something that has been emphasized in several studies [2, 4, 15, 21, 27]. However, to ensure favorable integration, FLMs need integration strategies that address intra-organizational, sociocultural, and professional development domains [15]. According to Kamau and colleagues, intraorganizational strategies should be tailored to a specific organization, such as policies that facilitated cultural and linguistic diversity within the workforce as well as collegial support. Further, organization-supported language learning can be used in strategies to develop sociocultural language and communication. Finally, FLMs have to employ professional development strategies that improve competence and professional development [15].

However, there is a lack of studies reporting on experiences of language development initiatives or interventions within the health care services [6, 9, 15]. Thus, the aim of the present study is to explore and describe the experiences of the language development intervention “Talk about Work” from the perspective of FLMs, language advocates and participating care staff.

## Method

### Design

The present study is explorative, inductive and qualitative in design. Data were collected through focus-group interviews (FGIs) to gain a deeper understanding of the informants’ experiences of a phenomenon [22]. On-going evaluation and research methods include a formative and process-oriented evaluation. The method allows researchers to study a language development intervention during its implementation, rather than afterwards. The method aims to improve the initiative or intervention in real time (Smith et.at. 2023).

### Setting

The study was conducted in a county in central Sweden consisting of eight municipalities and approximately 242,100 inhabitants, of whom about 23 percent were born abroad. The study was conducted in one of these eight municipalities, and that municipality has 37 NHs (operated either publicly or privately). Of these, nine are in rural areas and 28 are situated in urban areas. The median age for residents in Sweden moving into a NH is just over 86 years (The National Board of Health and Welfare [28]. The study was conducted in NHs caring for elderly residents aged approximately 70–100 years. Around 1,600 employees are working in the NHs, with an estimated 56% being foreign-born. Since 2020, the municipality has started several initiatives, hereafter called interventions, to support language development through a strategy entitled “Language development workplaces,” which is led by two process managers. One part of the overall strategy involves educating language assistants (1-day training) who are assigned to support their colleagues in communication at NHs, as well as educating language advocates (4-day training) to train the language assistants and support them at NHs.

#### The intervention talk about work

“Talk about Work” is a discussion series developed to strengthen language and professional competence within health and social care. The material includes a facilitator’s guide for discussion leaders, participant materials for those taking part in the discussions, a PowerPoint presentation for discussion leaders, and information material for FLMs. The material has been developed with the support of funding from the Swedish Public Employment Service and in close dialogue with workplaces. It aligns with the National Board of Health and Welfare’s *Language Proficiency in Elderly Care: Framework for Assessment and Development*. The eight topics for discussion available at the time of the present study included: verbal communication with older people, the difference between one-way and two-way communication, making telephone calls, verbal reporting between colleagues, confidentiality, written documentation, and understanding different cultures. The language assistants have been assigned to lead the intervention “Talk about Work” at NHs and to hold discussion workshops for their colleagues.

### Sample and procedure

Purposive sampling was used to include informants from publicly operated NHs in both urban and rural areas. Inclusion criteria were caring staff including language assistants, language advocates and FLMs at NHs where the language intervention “Talk about Work” was conducted from autumn 2023 to spring 2024. Four NHs were selected because they had an experienced FLM, who had been working as an FLM in the municipalities for more than 3.5 years (Table 1) and who reported having positive attitudes about testing the intervention. Further, the selected NHs did not have an ongoing staff shortage, and the caring staff should have attended at least one workshop in the initiative. No exclusion criteria were applied. Process managers from the municipality informed operation managers and FLMs at the NHs about the study. FLMs and language advocates responded via email, indicating their interest in participating in the study, while care staff, including language assistants, were asked by their FLMs and administrative staff to participate. The characteristics of the 28 informants who participated are presented in Table 1. Most were female and had worked at the NH for two to three years.

**Table 1:** Characteristics of the study population: first-line managers, language advocates and staff**,** including language assistants

Variable	First-line Managers *N* = 3	Language advocates *N* = 5	Staff, including language assistants *N* = 20
Age (years)^1^	43 (41–44)	42 (29–54)	39.5 (20–61)
**Gender** (n)			
Female	3	4	14
Male		1	6
**Level of education** (n)			
Primary school (< 9 years)			6^3^
High school (≥ 9–12 years)		5	9
Education at university level < 2 years			1
Education at university level > 2 years^2^	3		2
**Current working conditions**			
Full time	3	4	15
Part time		1	5
**Profession**			
Nursing assistant			10^2^
Associate nurse		5	9
Registered nurse	2^2^		
**Working in elderly care** (years)^1^	-	4.5 (1.5–38)	10 (5–24)
**Working as first-line manager** (years)^1^	7 (3.5–16)	-	-
**Number of years working at the current nursing home** (years)^1^	3.5 (3–7)	2 (1.5–9)^3^	3 (1.5–38)
**Country of birth** (n)			
Sweden	2	2	4
Afghanistan			4
Bangladesh			1
Eritrea		1	2
Ethiopia			1
Iraq			1
Iran	1		2
Marocco			2
Somalia		1	
Syria		1	
Tunis			1
Turkey			1
Uganda			1
**Languages they reported they could understand** (n)			
Swedish	3	5	20
English	3	3	8
Arabic		2	5
Aramaic		1	2
Bengali			1
Dari			3
Farsi	1		
French			1
Italian		1	1
Luo			1
Persian			5
Tigrinya		1	3
Turkish			2

^1^ Values are Median (Range). ^2^One missing value, ^3^Two missing values

### Data collection

Six FGIs were conducted in Swedish between June and August 2024. Four FGIs with care staff were held at NHs. One FGI with language advocates and one FGI with NH FLMs were held in secluded conference rooms familiar to the respondents. The interviews were led by a moderator (first author), and an assistant moderator (second author), who tape-recorded the sessions, took fieldnotes, and summarized the content at the end of each interview. Both authors are registered nurses with extensive experience in elderly care and qualitative interviewing. Fluent discussions and lively interaction were observed, and the FGIs lasted for 41–69 min [19]. An interview guide was developed by the research team in collaboration with the process managers from the municipality. It included questions covering informants’ sociodemographic data and experiences of leading and/or participating in the language development project "Talk about Work."

### Data analysis

Data analysis was performed following the method described for FGIs [19]. Collection and analysis of data proceeded simultaneously until it was judged that no new information was being added. The interviews were transcribed verbatim (by a professional secretary) and thereafter read through by the first author to get an overview of the content. The transcripts were exported to Open Code [13] for data organization. Thereafter, text strings related to the study aim were identified as meaning units, condensed and coded as close to the text as possible. The analysis continued by comparing the codes for similarities and differences and grouping codes with similar content into preliminary subcategories. The analysis process is presented in Table 2. The co-authors also checked and discussed the content of the codes in relation to the subcategories and categories until consensus was reached. The author’s pre-understanding was considered a strength as they were familiar with the setting and nursing home context. The authors also strived to actively bracket their pre-understanding of the phenomenon during the analysis and during the process had active discussion among them [22]. The Consolidated Criteria for Reporting Qualitative Research (COREQ) were followed [30].

**Table 2:** Example of the data analysis with meaning units, condensed text, codes and subcategories

Meaning units	Condensed text	Code	Subcategories
We’ve had meetings twice on language, on developing yourself. But we haven’t had language café here. As far as I know, outside the workplace meetings	We haven’t had language café outside the workplace meetings	Talking about communication at the workplace meetings	*Variations in implementation*
There are films you show at the beginning, and then you discuss … And there are really good questions. I mean the material is very good	There are films, discussion questions, very good material	There is good material you can use	*A good study material*
I have a friend at work who wants help, and then we can say, go to her (language assistant), because she can help you. They also need to make time for helping those who are having difficulties	Being able to tell a colleague to talk to a language assistant, because they can help. Language assistants need more time	Give language assistants more time	*Challenges along the way*
We exchanged a lot this lesson. I’ve been once, but it was valuable	Exchanged a lot during the lesson, it was valuable	Instructive	*Instructive and fun but also demanding*
In a better way, I think. Well, you … can you use a nice word. Maybe you go over. I mean, can you go, can you do	Talk with colleagues in a better way, use nicer words when you ask a colleague to do something	Participants changed how they spoke, collegially	*Positive changes after the intervention*
We really want to continue with it, because we need to move forward a bit, like how we communicate with everyone in the work group	Want to continue the initiative, need to develop communication in the work group	Continue using the material	*Plans for continuation* (of the language initiative)
It would be more fun if you could go for every topic and like see what you can learn	More fun if you can attend all the meetings	Wish to attend all meetings	*Requests for future language development*

### Ethical considerations

The Swedish Ethical Review Authority approved the study (Reg. no. 2020–03636 and 2022–01084-02), and the Helsinki Declaration [5] guided the procedure. Written informed consent was obtained from all informants prior to participation in the FGIs. Participation was voluntary, and informants were told they could withdraw from the study at any time. Anonymity between the informants in the focus groups cannot be guaranteed, which they were informed of, and they were instructed to share information only during the interviews. Transcripts were deidentified and confidentiality was ensured by pseudonymizing the data, which were handled and stored according to regulations.

## Results

The data analysis resulted in three categories and seven subcategories, which are presented in Table 3 and in the text below. The excerpts were selected to illustrate the lively discussions and are labelled using the numbers assigned to the members of each FGI.

**Table 3:** Table of categories and subcategories

Categories	Subcategories
The art of carrying out a language development initiative	*Variations in implementation* *A good study material* *Challenges along the way*
Mixed experiences and positive outcomes of the language intervention	*Instructive and fun but also demanding* *Positive changes after the intervention*
The way forward	*Plans for continuation (of the language initiative)* *Requests for future language development*

### The art of carrying out a language development intervention

Variations in implementation of the intervention “Talk about Work” were reported by both FLMs and language advocates, although all informants spoke positively about the study material. Different challenges during implementation of the intervention arose for FLMs, language advocates, and language assistants.

#### Variations in implementation

The language intervention was implemented in different ways at the NHs; some called it language café, some changed the name to “workshops” due to the sensitive nature of the word “language,” and others used regular staff meetings. In the present study, these gatherings will hereafter be referred to as “workshops.” At some NHs, all staff were welcome to attend the workshops, while other NHs focused on regular staff, thus excluding temporary workers, or management selected which care staff members would participate. The FLMs did not feel pressured to implement the intervention, but mentioned it was a request from municipal management. According to them, it was the FLMs’ function to enable the intervention, for example, by changing staff members' schedules to enable workshop participation. They reported that the language assistants could lead the workshops with support from an FLM or language advocates.

FLM 1: Everyone is welcome to take part in the language cafés. Then like I said, it was slow at first, nobody wanted to participate, but then when they were at the language café they thought “but that was fun, lots of laughing in the group, it was open.” And then we were very clear to say this is not a formal meeting, it’s a get-together (FGI FLM)

The language advocates prepared and held workshops and led discussions at workshops. They actively recruited participants and mentioned that, at first, few wanted to go to the workshops. Some staff did not recognize the need to participate in language development intervention, which made recruitment of participants difficult. Language advocates described helping their colleagues communicate in Swedish by assisting them with pronunciation and documentation, but it was difficult to approach a colleague who did not ask for help. A few mentioned that being able to support colleagues was rewarding. Language advocates felt the demands of educating 80 language assistants. Further, they did not feel trust from the FLMs at NHs and mentioned that FLMs need more knowledge about the intervention and available resources. During the implementation, some language assistants had stopped working, some did not want to lead the workshops, and workshops were cancelled due to staff shortages.

Language advocates 3: The thing is, when you approach a staff member, I’ve worked for 25 years, or 20 years, and say you have to come down to the language café, well, that’s a bit sensitive.

Language advocates 2: And the word language itself, it’s also charged.

Interviewer: Right. It becomes charged. But when we say discussion get-togethers, has that been easier?

Language advocates 3: Well, yes, in my experience. It’s much easier. (FGI Language advocates)

#### A good study material

All represented groups (FLMs, language advocates, staff including language assistants) reported good experiences of the study material “Talk about Work.” Topics such as palliative care, how to encounter residents and relatives, and answering and making phone calls were especially appreciated among staff. The study material was described as comprehensive, and short videos were used as an introduction for further discussions. Some FLMs mentioned how they had developed the material, making it more detailed regarding topics of food and encounters with relatives. Others mentioned possibilities to use the material in situations other than the workshops in “Talk about Work.” However, some FLMs and staff described needing something new, indicating they felt “finished” with the 8 topics.

FLM 4: Well, the material itself is very, very good and useful. The set-up has been carefully thought through. (FGI FLMs)

#### Challenges along the way

Several challenges were reported during implementation of the language intervention, as it involved hard work. For the FLMs, it was sometimes difficult to find motivated persons with the right competence (Swedish and pedagogic skills) to become language assistants, who were to plan and lead colleagues in group activities during the workshops. Another concern was selecting the right staff members to participate in the workshops. The FLMs' main concern was lack of time and financial resources, low staffing, and inadequate support from the operations managers. It was further mentioned that it was unclear who the language assistants were and what the municipality’s goals were with the language initiative in NHs.

The language advocates described needing more support from the FLMs. They needed more time to train more language assistants, and the FLMs had not sent people to the training. They also reported difficulties communicating with language assistants at the NHs when trying to follow up and support them.

Language advocate 3: We haven’t been given a chance.

Language advocate 2: No, not a real chance.

Language advocate 3: Other departments have been given four years. We got one year, you’ve worked maybe one, one and a half years.

Language advocate 1: Right, one and a half years. Then I had to leave. … No, well I mean, they …. It feels like they don’t take responsibility and don’t trust us, you know.

Interviewer: Yes, exactly.

Language advocate 3: The thing is, we want time to follow up with our language assistants. We haven’t had any. Last year we trained, trained, and emailed managers, called managers. That takes a lot of time. … To come in and give support and help. So we haven’t had that time, to take care of our language assistants.

Language advocate 2: And then the fact that we had to attend the language advocate training.

Language advocate 3: That takes time. Three months.

Language advocate 2: Yes, it took all my time. In three … well, what was it? September, October, November then I was done.

Language advocate1: And then it isn’t even used. We don’t train any language advocates, or language advocate teachers. (FGI Language advocates)

Participating care staff expressed limited confidence that the language assistants would ask questions, especially when the language assistants had limited Swedish language skills. Group dynamics within the staff group, hierarchies, and conflicts were mentioned as obstacles to effective workshops. Further, it was difficult for language assistants to correct others' Swedish language/pronunciation without hurting their feelings.

### Mixed experiences and positive outcomes of the language initiative

All represented groups (FLMs, language advocates and staff including language assistants) reported misunderstandings, due to limited Swedish language skills, happening occasionally at the NHs. Furthermore, they were aware of incident reports from relatives concerning meal situations and encounters with staff related to limited Swedish language skills. Further, all groups reported a combination of varied experiences from the intervention. FLMs and language advocates described problems of explaining the purpose of the intervention to caring staff, and problems realizing the intervention with limited resources were mentioned. However, beneficial outcomes of the language intervention were also expressed, along with disappointment when it did not proceed as intended.

#### Instructive and fun but also demanding

Some FLMs mentioned that the intervention turned out better than expected. Positive experiences of participating in the “Talk about Work” workshops were described, e.g., staff members had fun, learned something new, helped each other with language issues, and got to know each other better. However, for some FLMs, the intervention did not turn out as expected. The language assistants had low pedagogical competence, and they needed a lot of support to carry out the workshops. In the beginning, the staff needed many explanations of the intervention to understand the purpose of the workshops. Some mentioned that the climate at the workshops was not open and permissive.

Language advocates and language assistants mentioned that it was best to involve the FLMs and administrators at the NHs when recruiting participants to the workshops. Although it could be demanding to carry out the workshops, language assistants also said helping their colleagues was rewarding.

Staff perceived that, in an open climate, they dared to ask questions and could laugh when a language-related example was comical. Some reported receiving tips on how to handle difficult situations, and others mentioned the good discussions that followed after watching short videos.

Staff 4: We talked to each other. For example, she called me and I answered. I called, she answered.

Staff 2: And how you say, like …. How you treat. No, really it was fun.

Staff 4: Well, if a relative who, for example, she … a relative who called and wants to ask about her mother. Well, then she calls and I answer.

Staff 2: And then just the opposite.

Staff 4: Then the opposite. It was great fun.

Staff 2: Yes. It really was fun. (FGI 2 participating staff).

However, only being able to participate in one or two of the 8 workshops was another constraint for staff. Further, the workshops were too infrequent, and they did not get a lot done. At some NHs, only regular staff were invited to the workshops, and some reported that, for the intervention to be effective, both regular staff and temporary workers needed to participate in the workshops.

#### Positive changes after the intervention

The FLMs reported that one outcome of the intervention “Talk about Work” was a more open working climate where people dare to speak more freely and ask questions. Staff members experienced less shame if they said something wrong. After the intervention, they felt safer and more confident making phone calls in Swedish. They prepared themselves before each call and dared to contact nurses and relatives with more competence and self-confidence. Others felt more secure answering the phone.

Another positive result was when staff noticed a kinder way of talking to colleagues, as well as to residents and their relatives. Others mentioned kinder behavior/encounters with colleagues, as well as residents and their relatives.

Staff 6: But it happens many times at work, the group too. There are new temporary staff or maybe you’ve worked too long. You don’t dare talk when you’re sitting like that, the coffee break, like. You don’t dare talk, because you feel … What if I say it like this, then it’s wrong, maybe somebody will laugh at me, that I said something wrong.

Staff 2: That’s why we have these lessons, they make you stronger …

Staff 6: So it’s the way that … The thing I’m trying to work with, like. Talking … Just say what you think, like. Is it wrong … Wrong … Everybody makes mistakes.

Staff 2: You try.

Staff 6://Right, it turns out fine anyway, like. Then you’ve said something..//

Staff 2: Experience. You gain experience and I’ve noticed some of my colleagues changing their behavior with others. Especially with temporary staff. For example, I said give me the spoon, and she said, you can use a nice word. Please give me the spoon. (FGI 3 participating staff)

### The way forward

Informants offered several ideas for using the experiences of the intervention and the course material in the future. If work with language development at the NHs was to continue, the FLMs expressed the need for increased resources.

#### Plans for continuation (of the language intervention)

Despite challenges, informants had several ideas about how they planned to continue the language intervention at their workplaces. The FLMs mentioned setting aside time for language development at workplace meetings. They planned to repeat certain topics from the workshops and bring in new topics. Other FLMs said they would continue the intervention but at a different pace. They mentioned that it was important not to forget the material –– that it is a good starting point for various situations that may arise among staff. Some planned to continue with the “language café,” while others needed something new, a new forum and new material.

FLM 3: Yes, we’re continuing the workplace meetings. So when [name] came to see us, she came up with a topic she’d missed or hadn’t thought of, palliative, so we got a new topic there. So we add that. And then I think we might build out when the staff group changes, or certain topics we choose, but a bit more sporadically, not dropping it, but not so often. But it’s easy to forget, so we have to remind ourselves, and the group changes. So we’ll certainly continue, just not at the same pace. (FGI FLMs)

However, the language advocates reported that some FLMs needed more knowledge about the intervention and about the study material to continue the intervention. Another issue for them was being able to meet and follow up with the language assistants at the NHs to support them in their role.

Language advocates 2:The idea is that the language assistants should be able to run … Talk about Work meetings themselves. But we’re supposed to work with them to start things up and support them. Some may only need support at two meetings, other may need it all eight times. But the idea is that we let go and they take over themselves.

Language advocates 3:It’s different from person to person.

Language advocates 4: Yeah, … She asked us if she needed support … yes, please, if you just sit beside us and that’s good. … Then she said, next we’ll be by ourselves. (FGI Language advocates)

#### Requests for future language development

Several expressed their desire to continue the intervention in an effective way: The FLMs wanted more time to enable language assistants to lead workshops and help individual staff with language issues. They also requested resources in the form of funding, increased staffing at NHs, and improved competence among the language assistants. Some FLMs wanted to assess the staff's competence in Swedish and provide targeted language support based on individual needs.

FLM 1: No, but like I said, generally, there’s a lot more to be done with this work. And this Talk about Work, that’s only eight meetings, that’s nothing compared with the real work that needs to be done, and resources are needed. Staffing, that you are given the time, that you get support from management, you need to be engaged while it’s happening. So the desire is that … well, like I said, time-wise, resource-wise, because there’s a lot of work to do. We need to sit down, go through, highlight and work on what needs to be done. (FGI FLM)

The language advocates wished they had more communication/contact with the other language advocates and more network meetings with all language assistants to learn from each other. Staff reported wanting to attend all workshops and for the workshops to be scheduled more often. Additionally, they stated that it would be better if those who held the workshops were Swedish-born or colleagues whose spoken Swedish was fluent and modelled good pronunciation. Furthermore, staff said they could develop their Swedish skills in different ways, e.g., by talking more with colleagues and residents at work. They also suggested they could read Swedish books and watch Swedish films and television using different language applications.

Staff 4: So I mean. It’s not good. If an advocate is from another country and doesn’t speak Swedish very well. (FGI 2 participating staff including Language advocate)

## Discussion

The present study looked at an intervention to enhance Swedish language skills from the perspective of FLMs, language advocates and care staff at NHs involved in the intervention “Talk about Work.” All represented groups appreciated the educational material, and the FLMs thought it would also be useful in future educational efforts. Swedish language skills were regarded as a sensitive issue to address at work, and the intervention had created an openness to speaking about language competence. According to the FLMs, one challenge of the intervention for FLMs was finding staff with sufficient competence to take on the role of language assistants who lead workshops at the NHs. Language advocates wished they could have closer communication with FLMs to plan workshops and to support language assistants. Caring staff appreciated the workshops and highlighted the importance of letting temporary staff attend the workshops as well as of being able to participate in all workshops.

Carrying out a language development intervention in clinical settings is not an easy task. Maybe this is one reason for the lack of studies on the experience of language development interventions in the health care services [6, 9, 15]. The present study is one of very few reporting results from a language development intervention targeting care staff in NHs. The findings show that the intervention “Talk about Work” and its material were considered appropriate for and were well received by FLMs, language advocates, and participating care staff, including language assistants. According to Kamau and colleagues [15], strategies that focus on intra-organizational, sociocultural and professional development must be used to integrate foreign-born care workers. The “Talk about Work” intervention used all three integration strategies. The municipal management and FLMs designed policies tailored to the specific organization of the NHs, which facilitated cultural and linguistic diversity in the workforce as well as peer support. Furthermore, organizationally supported language learning and strategies to develop sociocultural language and communication The present study reports on how the intervention was implemented in various ways suited to each NH and their respective FLMs. To enhance implementation and the effects of different interventions, it is important to consider what is suitable for each NH and their respective FLMs. In order to make changes that suite the NHs and FLMs, it is also important to allow FLMs to share their experiences [6, 10]. The framework for promoting research implementation in health care (PARIHS) also emphasizes that the implementation process should be carried out in close collaboration with adopters (FLMs, language advocates and care staff), implementers (process managers and operation managers) and maintainers (The Health and Social Care administration at the municipality). According to the PARISH framework, there are joint effects between strong evidence, a favorable context and the facilitating factors required for successful implementation in clinical practice [17].

However, there were challenges along the way. Both FLMs and language advocates described difficulties in finding motivated persons with the right competence (Swedish and pedagogic skills) to become language advocates or assistants, as well as in selecting the right care staff to participate in the workshops. Also, group dynamics within the staff group, hierarchies, and conflicts were mentioned as obstacles to effective workshops, a finding that has been reported in other studies [6, 10]. According to [23], successful implementation requires that the large group, which is slower to adopt an intervention, actually do so. To achieve this, informal leaders and stakeholders must be persuaded. One should not waste time trying to get everyone on board; instead, "early adopters"—as in the present study—can be utilized to test an intervention, evaluate it, and at a later stage present proposals for various implementation methods to all NHs.

Experiences of the “Talk about Work” intervention were mixed. Although language was a sensitive or “charged” issue in the workplace, the results indicated positive experiences with the intervention, such as an openness to discussing language proficiency within multicultural staff groups at NHs. Further, the intervention led to kinder and more competent encounters between colleagues and residents and their relatives. Similar experiences are also reported in another study [18]. This indicates that, to improve language and communication skills among caring staff in multi-cultural NH contexts, there is a need to find staff with sufficient competence to lead language development interventions as well as for long-lasting resources required to implement them. This is not a quick-fix.

In the category, the way forward, after the intervention” Talk about Work,” the participants expressed a need to continue the language intervention. The FLMs reported needing a plan for how to carry on. All participants also wanted to see future language development interventions. Other studies have also highlighted the need for varied educational material and approaches [6, 10].

The strengths and limitations of the present study also need to be addressed when interpreting the findings. One major strength was the explorative and inductive qualitative design, which allowed for deeper description and evaluation in real-time of the intervention "Talk about Work" and its implementation process (Smith et al., 2023)**.** The study’s rigor was enhanced through the collaboration between researchers and the two process managers on the strategy entitled “Language development workplaces.” They worked closely together formulating the research aim and questions as well as the methods, including the inclusion of study participants and development of the interview guides. Elderly care management in the municipality and the two process managers were continuously informed of the results through oral and written rapid reports, allowing the intervention to be adjusted.

Regarding qualitative research findings, a study’s trustworthiness, including credibility, dependability, confirmability, and transferability, are essential in informing policy decisions and improving the service provision at, for example, NHs. The present findings’ trustworthiness was strengthened by achieving credibility, which was accomplished by collecting the data through FGIs, thus providing a deeper understanding of the informants' experiences of the "Talk about Work" intervention. Credibility was also strengthened by triangulation through the inclusion of three groups (FLMs, language advocates and care staff) in six separate FGIs. Further, credibility was strengthened by the significant number of statements made in the FGI discussions and by reporting direct excerpts from the participants’ responses, thus strengthening the study’s dependability. Dependability was also strengthened by following the method described for FGIs [19] and describing the research process thoroughly, according to the COREQ [30]. Confirmability was strengthened by the authors' experiences in qualitative research, which also included taking into account their pre-understandings. Further, confirmability was strengthened by their collaborative analysis process in which they read the transcripts, checked and discussed the content of the codes in relation to the subcategories and categories until consensus was reached [22].

However, certain limitations should also be highlighted. In FGIs there is always a risk that participants will influence each other’s opinions. Further, participants’ responses may have been influenced by hierarchical relationships within the groups, potentially leading to socially desirable answers. However, to reduce this risk and create a safe environment for open and authentic expression of experiences, the FGIs sessions were conducted within homogeneous professional groups [19]. Moreover, a limitation was the selection procedure, although it could be argued to be a reasonable feasibility-driven choice, it may have resulted in a selection and social desirability bias, which could jeopardies the transferability of the results. Also, the present study was conducted in Sweden, which may limit its transferability to other contexts, where organizational and professional structures may differ. Thus, whether the findings can be applied to the reader’s context will be left to the reader to judge.

Notwithstanding potential limitations in credibility and transferability, the present findings remain significant given that the study fills a gap in knowledge with regard to experiences of language development interventions within NHs [6, 9, 15].

## Conclusion

The present findings show that the intervention “Talk about Work” was considered appropriate for and was well received by the participating FLMs, language advocates, and caring staff. Moreover, they indicate that it is important to find staff with sufficient competence to lead language development interventions as well as to provide the necessary resources to implement them. Further, more studies are needed that offer insights into which interventions, aimed at improving communication skills among foreign-born NH staff, are most effective and feasible.

## Supplementary Information

Supplementary Material 1.

## Data Availability

The datasets are not available from the corresponding author on request as the authors have promised to protect participants’ privacy and confidentiality.

## Abbreviations

FLMs: First-line managers
NHs: Nursing Homes
FGIs: Focus-group interviews
